# 17.6% of patients in a German cohort with exocrine pancreatic cancer were diagnosed with a genetic tumor syndrome—a case for universal genetic testing?

**DOI:** 10.1016/j.esmogo.2025.100218

**Published:** 2025-08-08

**Authors:** D. William, M. Bermúdez, A. Kübler, C. Kahlert, M. Distler, J. Weitz, S. Uhrig, M. Fröhlich, B. Hutter, D. Aust, G. Baretton, P. Wimberger, K. Kast, C. Meisel, L. Gieldon, J. Porrmann, J. Wagner, M. Arlt, M. Franke, J. Fischer, K. Hackmann, S. Kreutzfeldt, A. Mock, C.E. Heilig, D.B. Lipka, M.-V. Teleanu, R.F. Schlenk, B. Brors, D. Hübschmann, N. Paramasivam, D. Richter, K. Beck, K. Pfütze, I. Buchhalter, W. Weichert, T. Herold, K. Spiekermann, P.J. Jost, U. Keilholz, F. Klauschen, S. Bauer, J.T. Siveke, T. Kindler, M. Boerries, A.L. Illert, M. Bitzer, K. Schulze-Osthoff, P. Schirmacher, A. Stenzinger, P. Horak, C. Heining, G. Folprecht, S. Fröhling, H. Glimm, E. Schröck, A. Jahn

**Affiliations:** 1 Institute for Clinical Genetics, University Hospital Carl Gustav Carus at TUD Dresden University of Technology and Faculty of Medicine of TUD Dresden University of Technology, Dresden; 2 ERN GENTURIS, Hereditary Cancer Syndrome Center Dresden, Dresden; 3 National Center for Tumor Diseases (NCT), NCT/UCC Dresden, a partnership between DKFZ, Faculty of Medicine and University Hospital Carl Gustav Carus, TUD Dresden University of Technology, and Helmholtz-Zentrum Dresden-Rossendorf (HZDR), Dresden; 4 German Cancer Consortium (DKTK), Dresden; 5 German Cancer Research Center (DKFZ), Heidelberg; 6 Core Unit for Molecular Tumor Diagnostics (CMTD), National Center for Tumor Diseases (NCT), NCT/UCC Dresden, a partnership between German Cancer Research Center (DKFZ), Faculty of Medicine and University Hospital Carl Gustav Carus, TUD Dresden University of Technology and Helmholtz-Zentrum Dresden-Rossendorf (HZDR), Dresden; 7 Department for Visceral, Thoracic and Vascular Surgery, University Hospital Carl Gustav Carus at TUD Dresden University of Technology and Faculty of Medicine of TUD Dresden University of Technology Dresden, Dresden; 8 Computational Oncology Group (CO), Molecular Precision Oncology Program (MPOP), German Cancer Research Center (DKFZ), Heidelberg; 9 National Center for Tumor Diseases (NCT), NCT Heidelberg, a partnership between DKFZ and Heidelberg University Hospital, Heidelberg; 10 Institute of Pathology, University Hospital Carl Gustav Carus at TUD Dresden University of Technology and Faculty of Medicine of TUD Dresden University of Technology, Dresden; 11 Department of Gynecology and Obstetrics, University Hospital Carl Gustav Carus at TUD Dresden University of Technology and Faculty of Medicine of TUD Dresden University of Technology, Dresden; 12 Division of Translational Medical Oncology, German Cancer Research Center (DKFZ), Heidelberg; 13 German Cancer Consortium (DKTK), Core Center Heidelberg, Heidelberg; 14 Institute of Human Genetics, Heidelberg University, Heidelberg; 15 Department of Internal Medicine V, Heidelberg University Hospital, Heidelberg; 16 National Center of Tumor Diseases Trial Center, German Cancer Research Center and Heidelberg University Hospital, Heidelberg; 17 Computational Oncology Group, Molecular Precision Oncology Program, NCT Heidelberg and DKFZ, Heidelberg; 18 Division of Applied Bioinformatics, DKFZ, Heidelberg; 19 Pattern Recognition and Digital Medicine Group, Heidelberg Institute for Stem Cell Technology and Experimental Medicine (HI-STEM gGmbH), Heidelberg; 20 Department of Translational Medical Oncology, National Center for Tumor Diseases (NCT), NCT Dresden, a partnership between DKFZ, Faculty of Medicine and University Hospital Carl Gustav Carus, TUD Dresden University of Technology, and Helmholtz-Zentrum Dresden-Rossendorf (HZDR), Dresden; 21 Center for Personalized Oncology, University Hospital Carl Gustav Carus at TUD Dresden University of Technology and Faculty of Medicine of TUD Dresden University of Technology, Dresden; 22 Sample Processing Laboratory, German Cancer Research Center (DKFZ) and National Center for Tumor Diseases (NCT) Heidelberg, Heidelberg; 23 Omics IT and Data Management Core Facility, German Cancer Research Center (DKFZ) and German Cancer Consortium (DKTK), Heidelberg; 24 School of Medicine, Institute of Pathology, Technical University of Munich, Munich; 25 Department of Medicine III, University Hospital, Ludwig Maximilians University Munich, Munich; 26 Department of Hematology, Oncology, and Stem-Cell Transplantation, Munich Hospital Bogenhausen, Munich Municipal Hospital Group, Munich, Germany; 27 Division of Oncology, Department of Internal Medicine, Medical University of Graz, Graz, Austria; 28 Charité Comprehensive Cancer Center, Charité-Universitätsmedizin Berlin, Corporate Member of Freie Universität Berlin and Humboldt-Universität zu Berlin, Berlin; 29 German Cancer Consortium (DKTK), partner site Berlin, and German Cancer Research Center (DKFZ), Heidelberg; 30 Institute of Pathology, Ludwig Maximilian University of Munich, Munich; 31 BIFOLD-Berlin Institute for the Foundations of Learning and Data, Berlin; 32 Department of Medical Oncology and Sarcoma Center, West German Cancer Center, University Duisburg-Essen, Medical School, Essen; 33 DKTK, Partner Site Essen, German Cancer Consortium (DKTK), Heidelberg; 34 Bridge Institute of Experimental Tumor Therapy (BIT) and Division of Solid Tumor Translational Oncology (DKTK), West German Cancer Center, University Hospital Essen, University of Duisburg-Essen, Essen; 35 University Cancer Center and Department of Internal Medicine III, University Medical Center Mainz, Mainz; 36 TRON-Translational Oncology, University Medical Center, Johannes Gutenberg University Mainz, Mainz; 37 DKTK, Partner Site Frankfurt/Mainz, a partnership between DKFZ and University Medical Center Mainz, Mainz; 38 Institute of Medical Bioinformatics and Systems Medicine, Medical Center—University of Freiburg, Faculty of Medicine, University of Freiburg, Freiburg; 39 German Cancer Consortium (DKTK), Partner Site Freiburg, a partnership between DKFZ and Medical Center—University of Freiburg, Freiburg; 40 Department of Internal Medicine I, Division of Hematology, Oncology and Stem Cell Transplantation, University Medical Center Freiburg, Freiburg im Breisgau; 41 Department of Internal Medicine III, Klinikum Rechts der Isar, School of Medicine, Technical University of Munich, Munich; 42 Department of Internal Medicine I, University Hospital Tübingen, Eberhard Karls University of Tübingen, Tübingen; 43 Department of Molecular Medicine, Interfaculty Institute for Biochemistry, Eberhard Karls University of Tübingen, Tübingen; 44 Institute of Pathology, Heidelberg University Hospital, Heidelberg; 45 Department of Medicine I, University Hospital Carl Gustav Carus at TUD Dresden University of Technology and Faculty of Medicine of TUD Dresden University of Technology, Dresden, Germany

**Keywords:** familial pancreatic cancer, germline testing, hereditary cancer, genetic tumor syndromes, targeted therapy, pancreatic adenocarcinoma, parallel germline and tumor testing

## Abstract

**Background:**

Yields for (likely) pathogenic germline variants (PGVs) in cancer predisposition genes (CPGs) in pancreatic cancer (PCA) cases range from 5% to 10% in initial literature to 15% to 20% in recent literature. PGVs can impact therapy recommendations and cancer surveillance for individuals and families.

**Patients and methods:**

We retrospectively evaluated prospective cancer predisposition testing in 125 patients with exocrine PCA from a single-center clinical genetics clinic (*n* = 41) and a multicenter precision oncology program (*n* = 84) within 64 genes, including 14 established PCA risk genes. Associations with clinical and somatic molecular parameters, as well as therapy recommendations, were assessed.

**Results:**

PGVs were identified in 21.6% of patients (*n* = 27/125) across 14 CPGs. A genetic tumor syndrome was diagnosed in 17.6% of patients (*n* = 22/125). Existing inclusion criteria for germline testing [European Society for Medical Oncology (ESMO), American Society of Clinical Oncology (ASCO), National Comprehensive Cancer Network (NCCN)] would have missed up to 23.8% of PGV carriers (*n* = 5/21). Age of onset was not associated with PGV yield. A meta-analysis of 10 other PCA cohorts showed a median PGV yield of 14.1%.

In a precision oncology program, 10.7% (*n* = 9/84) of PCA patients received treatment recommendations supported by PGVs. Genetic testing was carried out on relatives of 73.3% of PGV-positive patients (*n* = 11/15), with one family demonstrating PGV confirmation in 7 of 13 tested relatives.

**Conclusions:**

These findings support ASCO and NCCN recommendations for germline testing in all PCA patients. We suggest offering large-panel genetic diagnostics early in clinical management, regardless of clinical parameters, with ongoing evaluation and adjustment of the gene panel.

## Highlights

• Across two German cohorts, 17.6% of pancreatic cancer patients were diagnosed with a genetic tumor risk syndrome.
• Strict germline testing criteria would have missed up to 23.8% of pathogenic germline variant carriers.
• Two-thirds of pathogenic germline variants informed treatment; 73% led to cascade testing, identifying 18 carriers.
• A meta-analysis of 11 PCA cohorts shows pathogenic germline variants in ∼14%—higher than the previously estimated 10%.
• Pathogenic germline variants in pancreatic cancer are under-recognized—universal testing and multidisciplinary care is needed.

## Introduction

Exocrine pancreatic cancer (PCA) is the third most common gastrointestinal cancer and is projected to become the second leading cause of cancer-related deaths in Western countries by 2030.^1^^,^^2^ The disease typically peaks in incidence at 65-69 years for men and 75-79 years for women, with a poor overall 5-year survival rate of only 8%.^3^ Despite general advances in cancer treatment, targeted therapy options for PCA patients are scarce. The poly (ADP-ribose) polymerase (PARP) inhibitor olaparib was approved as maintenance therapy for germline *BRCA1/2*-mutated, platinum-sensitive metastatic PCA, based on the POLO trial, which demonstrated prolonged progression-free survival (PFS) in these patients, and is under investigation as a potential treatment of PCA patients with (likely) pathogenic germline variants (PGVs) in other DNA damage repair-associated genes.^4^^,^^5^

Early detection of PCA and identification of high-risk individuals are crucial for improving the overall survival rate.^6^ The genetic risk factors for PCA are not yet fully understood. A proportion of PCA cases are associated with PGVs, particularly in *BRCA1/2* in the European population (5%-7%).^6^^,^^7^ The increased risk may manifest as familial PCA (FPC), which is defined as having at least two first-degree relatives with PCA, without other hereditary (tumor) syndromes associated with increased PCA risk.^7^

It is important to note that the absence of familial PCA clustering does not exclude a PGV in PCA patients. Thus, diagnostic testing for PGVs in PCA patients may be of benefit to patients and their families at risk for familial PCA or for other genetic tumor syndromes.

Screening protocols involving endoscopic ultrasound or magnetic resonance imaging have shown benefits in resectability and survival.^8^^,^^9^ Enhancing our understanding of genetic risk factors and implementing effective screening protocols can significantly impact early detection and management of PCA.

Despite the therapeutic relevance and potential for cancer screening in patients and high-risk individuals, routine germline testing for all PCA patients has not yet been universally implemented, with varying guideline recommendations across organizations. The National Comprehensive Cancer Network (NCCN) and the American Society of Clinical Oncology (ASCO) recommend germline testing for all PCA patients, regardless of family history or disease stage.^10^^,^^11^ This recommendation is based on recent research showing that germline mutations can occur at similar rates in PCA patients with or without a family history of cancer.^12^

The European Society for Medical Oncology (ESMO) has more restrictive recommendations. They suggest genetic testing only for patients with metastatic PCA who are eligible for platinum-based chemotherapy or PCA patients who meet FPC criteria.^13^ These differing recommendations highlight the ongoing debate in the medical community regarding the optimal approach to germline testing in PCA patients.

Furthermore, there is a lack of consensus regarding which specific genes should be included in genetic testing panels for PCA patients.

Cancer predisposition genes (CPGs) associated with increased PCA risk encompass several categories. The genes primarily associated with hereditary breast and ovarian cancer (HBOC) include *ATM*, *BRCA1/2*, and *PALB2*. Lynch syndrome (LS) genes comprise *MLH1*, *MSH2/6*, *PMS2*, and *EPCAM*. Other genes associated with increased PCA risk are *CDKN2A* [familial atypical multiple mole melanoma (FAMMM)], *STK11* [Peutz–Jeghers syndrome (PJS)], *TP53* [Li–Fraumeni syndrome (LFS)], *APC* [familial adenomatous polyposis (FAP)], and various hereditary pancreatitis genes.^7^^,^^14^ The yield of PGVs in PCA patients varies widely, ranging from 4% to nearly 20%, depending on the gene panel used, variability in variant evaluation, and patient selection criteria.^15^, ^16^, ^17^, ^18^, ^19^, ^20^, ^21^, ^22^, ^23^

In this study, we conducted a retrospective analysis of PGVs in 64 CPGs across two German PCA cohorts. The cohorts, comprising 125 patients in total, were drawn from a clinical genetics clinic and a precision oncology program. Our analysis revealed that 17.6% of patients (*n* = 22/125) in the combined cohort carried a PGV in a dominant CPG. We evaluated various inclusion criteria for germline testing and examined the associations between PGVs and clinical and molecular parameters, as well as their clinical implications. Our findings aim to inform and improve genetic testing strategies and personalized care for PCA patients.

## Materials and methods

### Patients and clinical data

The retrospective analysis included 125 patients with PCA diagnosed between 2013 and 2021. Eighty-four were part of the National Center for Tumor Diseases (NCT)/German Cancer Research Center (DKFZ)/German Cancer Consortium (DKTK)-Molecularly Aided Stratification for Tumor Eradication (MASTER) precision oncology program (MASTER cohort), which focuses on advanced cancers in young patients (<51 years) and rare subtypes, providing molecular profiling, therapy recommendations, and genetic counseling.^24^^,^^25^ Forty-one patients were referred to genetic counseling at Dresden’s Institute for Clinical Genetics (Dresden cohort), primarily for high-risk screening or hereditary cancer evaluation. One Dresden cohort patient later joined MASTER. Patients with hereditary pancreatitis and endocrine PCA were excluded. Family cancer histories were documented for 64 patients. Protocols followed the Declaration of Helsinki, with ethics approvals from Heidelberg (S-206/2011, MASTER) and Dresden (EK-495112022) institutions, and written consent obtained for anonymized data use. Clinical and familial cancer data were extracted from medical records (Supplementary Table S1, available at https://doi.org/10.1016/j.esmogo.2025.100218).

### Next-generation sequencing

DNA from Dresden cohort patients was isolated from blood and ultrasonically sheared to 180-250 bp fragments (Covaris, LLC, Woburn, MA), with size verification via Fragment Analyzer (Agilent Technologies, Santa Clara, CA). Libraries were prepared using the TruSeq Nano DNA Kit and sequenced on a NextSeq500/550 (2 × 150 bp) after targeting 94 or 113 CPGs (TruSight Cancer panels, Illumina Inc., San Diego, CA). Fastq files were aligned to hg19 using Qiagen Biomedical Genomics Workbench 5.0 (Qiagen, Hilden, Germany), with variants detected via CLC BMW’s low-frequency tool (minimum variant allele frequency of 10%, target regions only). The MASTER cohort provided germline/somatic variant data and mutational signatures from tumor/control whole exome sequencing/whole genome sequencing.^24^, ^25^, ^26^

### Bioinformatics gene panel

The available sequencing data (Supplementary Table S1, available at https://doi.org/10.1016/j.esmogo.2025.100218) were filtered for variants in a core set of 14 genes recommended by ASCO and NCCN for genetic testing in PCA patients (*APC, ATM, BRCA1/2, CDKN2A, EPCAM, MLH1, MSH2/6, MUTYH, PALB2, PMS2, STK11, TP53*) and 50 additional hereditary cancer-associated genes (*ALK, BAP1, BARD1, BLM, BMPR1A, BRIP1, CBL, CDH1, CDK4, CDKN1B, CHEK2, CYLD, DICER1, EXT1, EXT2, FH, FLCN, KIT, MAX, MEN1, MET, NBN, NF1, NF2, PHOX2B, POLD1, POLE, PRKAR1A, PTCH1, PTEN, RAD51C, RAD51D, RB1, RECQL4, RET, RHBDF2, RUNX1, SDHA, SDHB, SDHC, SDHD, SMAD4, SMARCA4, SMARCB1, SUFU, TMEM127, TSC1, TSC2, VHL, WT1*).^7^^,^^10^^,^^11^^,^^25^ Of note, *BARD1* was not included in the TruSightCancer94 panel (Supplementary Table S1, available at https://doi.org/10.1016/j.esmogo.2025.100218). Genes associated with hereditary pancreatitis were excluded. Variant classification was carried out in accordance with the standards and guidelines of the American College of Medical Genetics and Genomics and the Association for Molecular Pathology (ACMG-AMP) and further ClinGen specifications.^27^^,^^28^

### Inclusion criteria for germline testing of PCA patients

Medical records and family cancer histories were reviewed to identify patients meeting germline testing criteria. Families fulfilled criteria for familial pancreatic cancer (two or more first-degree relatives or one first-degree relative plus another relative with PCA), familial breast/ovarian cancer, or LS (Amsterdam-II), as detailed in Supplementary Table S1, available at https://doi.org/10.1016/j.esmogo.2025.100218.^7^^,^^29^^,^^30^ No cases met criteria for PJS, FAMMM, or LFS.^31^, ^32^, ^33^ German guidelines restricting germline testing to *BRCA1/2* in metastatic PCA patients for PARP inhibitors were excluded from this analysis.

### Statistics

To assess whether two population proportions significantly differ on a single, categorical characteristic, the z-score test for two population proportions was used. The Kolmogorov–Smirnov test was used to determine if a sample distribution matches the characteristics of a normal distribution. For comparisons of numerical variables between two groups the *t*-test for two independent means was used.

## Results

### Likely pathogenic or pathogenic germline variants were identified in 21.6% (27/125) of patients in two cohorts

The study included 125 PCA patients: 84 from the multicenter MASTER precision oncology program and 41 from the Dresden genetics clinic (Table 1, Supplementary Table S1, available at https://doi.org/10.1016/j.esmogo.2025.100218).^24^^,^^25^ The MASTER cohort had a younger median age of onset (45.5 years versus 60.0 years) due to its inclusion criterion of age <51 years, while the Dresden cohort had more prior cancer diagnoses (31.7% versus 6.0%, *P* < 0.001) and greater family cancer history availability (97.6% versus 28.6%). Dresden patients more often had a first-degree relative with cancer (82.9% versus 37.5%, *P* < 0.001) or PCA (50.0% versus 8.3%, *P* < 0.001). Germline variant analysis of 64 CPGs was carried out for most patients, except for 20 analyzed with the TruSight Cancer 94 panel, which excluded *BARD1*. Tumor sequencing was available for most MASTER patients (95.2% versus 7.3% in Dresden, Table 1).

**Table 1:** Patient characteristics

	Total cohort *N* = 125 (%)	Dresden cohort *n* = 41 (%)	MASTER cohort *n* = 84 (%)
Sex, *n* (%)			
Male	61 (48.8)	18 (43.9)	43 (51.2)
Female	64 (51.2)	23 (56.1)	41 (48.8)
Age, years			
Median	48	60	45.5
Range	15-85	34-85	15-80
Previous cancer diagnosis, *n* (%)			
Yes	18 (14.4)	13 (31.7)	5 (6.0)
No	106 (84.4)	27 (65.9)	79 (94.0)
N/A	1 (0.8)	1 (2.4)	—
Pedigree information available, *n* (%)			
Yes	64 (51.2)	40 (97.6)	24 (28.6)
No	61 (48.8)	1 (2.4)	60 (71.4)
History of cancer in at least one FDR, *n* (%)			
Yes, any entity including PCA	43 (34.4)	34 (82.9)	9 (10.7)
Yes, PCA	22 (17.6)	20 (48.8)	2 (2.3)
No	21 (16.8)	6 (14.6)	15 (17.9)
Unknown	61 (48.8)	1 (2.4)	60 (71.4)
Tumor sequencing available, *n* (%)			
Yes	88 (70.4)	4 (9.8)	84 (100.0)
No	37 (29.6)	37 (90.2)	0 (0.0)

FDR, first-degree relative; N/A, not available; PCA, pancreatic cancer.

**Figure 1:**
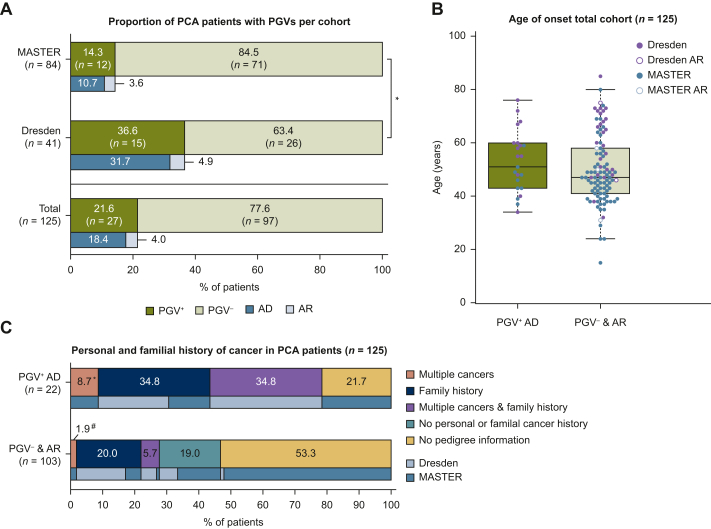
**Characterization of pancreatic cancer (PCA) patients across cohorts.** (A) Percentage of pathogenic germline variant (PGV) carriers in all patients (*n* = 125), the MASTER cohort (*n* = 84), and the Dresden cohort (*n* = 41). blue bars: patients with PGVs in autosomal dominant (AD) cancer predisposition genes (CPGs); light blue bars: patients with PGVs in autosomal recessive (AR) CPGs. ∗*P* < 0.01. (B) Age of onset in PGV-negative and AD-PGV-positive PCA patients (*n* = 125), Green bar: patients with PGVs in AD CPGs (*n* = 22), light green bar: patients with no identified PGVs (*n* = 98) or PGVs in AR CPGs (*n* = 5); Blue dots: MASTER patients; purple dots: Dresden patients. (C) Previous cancer diagnoses and family history of cancer in patients with PGVs in AD CPGs (*n* = 22, upper bar) and patients with no identified PGVs (*n* = 98) or PGVs in AR CPGs (*n* = 5, lower bar), Light blue bars: patients from Dresden, blue bars: patients from MASTER. ∗One patient with multiple cancers and unavailable pedigree information. ^#^Two patients with multiple cancers and unavailable pedigree information.

Germline analysis identified 33 PGVs in 27/125 patients (21.6%), with a significantly higher rate in the Dresden cohort (36.6% versus 14.3%, *P* = 0.0045, Figure 1A). Most PGVs (81.8%, *n* = 27/33) were in autosomal dominant CPGs, diagnosing genetic tumor syndromes in 22/125 patients (17.6%, Supplementary Table S2, available at https://doi.org/10.1016/j.esmogo.2025.100218). Only heterozygous PGVs in autosomal recessive CPGs were detected. There was no significant difference in median age of onset between PGV carriers and noncarriers (51.0 years versus 47.0 years, *P* = 0.16, Figure 1B). Among patients with prior cancer diagnoses, 55.6% (*n* = 10/18) had a PGV in an autosomal dominant CPG, as did 37.2% (*n* = 16/43) with a family cancer history (Figure 1C). Pedigree data were unavailable for 48.8% of patients (*n* = 61/125).

### Up to 23.8% of PGV carriers would have been missed by applying strict guidelines for germline testing in 64 PCA patients with available clinical information

**Figure 2:**
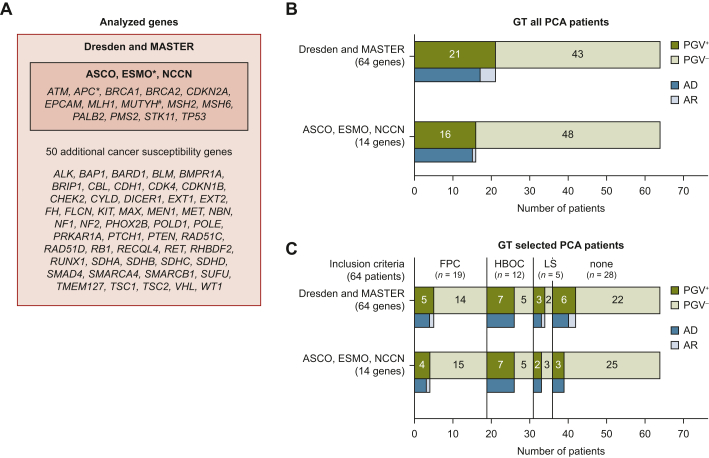
**Analyzed gene panels and fulfillment of genetic testing (GT) criteria by pancreatic cancer (PCA) patients**. (A) List of all tested genes (64 genes, red box) including PCA risk genes recommended for testing by European Society for Medical Oncology (ESMO), American Society of Clinical Oncology (ASCO), and National Comprehensive Cancer Network (NCCN) (14 genes, blue box). ESMO∗ recommends testing at least *BRCA1/2* for metastatic PCA and 14 CPGs in cases with familial clustering. ∗*APC* is only included in the ASCO and ESMO recommendations, ^#^*MUTYH* is only included in the ESMO recommendation.^10^^,^^13^^,^^34^ (B) Number of patients with PGVs in 64 genes (upper bar) or 14 genes (lower bar, ESMO, ASCO, NCCN recommendation) of all PCA patients. (C) Analysis of 64 genes or 14 genes of PCA patients who met the ESMO GT inclusion criteria for either familial PCA (FPC), Lynch syndrome (LS), or hereditary breast or ovarian cancer (HBOC), and patients not meeting any of the criteria (‘none’).

International guidelines recommend germline testing for all PCA patients, with varying gene sets. ASCO and NCCN suggest testing 13 PCA risk genes, while ESMO advises *BRCA1/2* testing for metastatic PCA and expanding to 14 genes for familial clustering (Figure 2A).^10^^,^^11^^,^^13^^,^^34^ In a group of 64 patients with sufficient clinical data, analysis of 64 genes identified 26 PGVs in 21/64 patients (32.8%). Testing only the 14 recommended genes would detect PGVs in 16/64 patients (25.0%), missing additional PGVs in *CHEK2*, *SDHB*, *BARD1*, *WT1*, and *NBN* (Figure 2B, Supplementary Table S1, available at https://doi.org/10.1016/j.esmogo.2025.100218). Applying germline testing criteria (HBOC, LS, FPC) would test only 36/64 patients (56.3%), identifying PGVs in 15/36 (41.7%), while missing PGVs in *CHEK2* and *NBN*.^7^^,^^29^^,^^30^ Among the remaining 28 patients not meeting criteria, 6 had PGVs in autosomal dominant CPGs (*PALB2*, *BRCA2*, *BARD1*, *SDHB*, *WT1*) and 2 in *NBN* (Figure 2C). Testing only *BRCA1/2* as per ESMO guidelines would identify 6/64 patients (13.3%) but miss 9 others with PGVs in autosomal dominant or autosomal recessive CPGs.^13^

### Two-thirds of PGVs supported treatment recommendations and led to cascade testing in 73% of patients with PGVs

**Figure 3:**
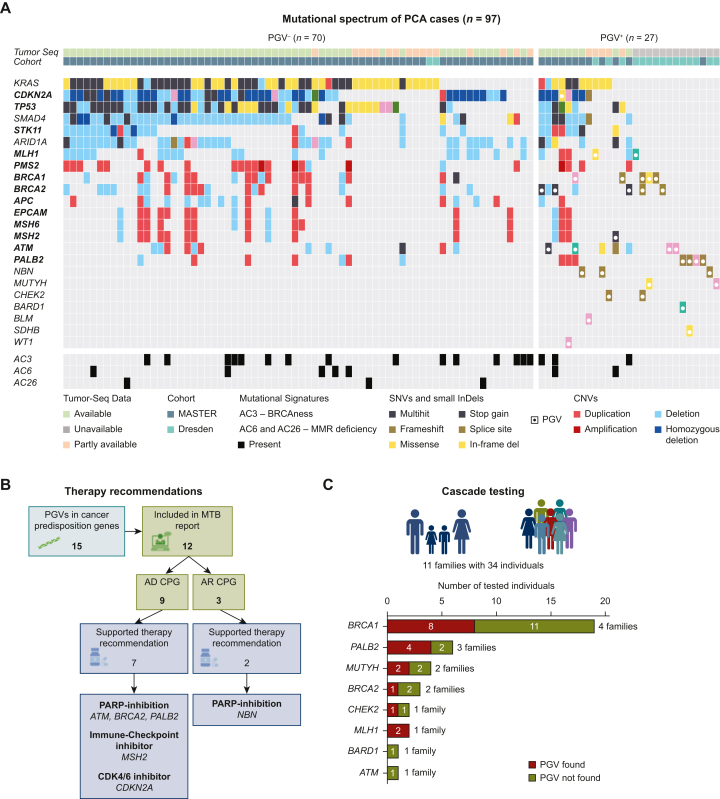
**Molecular analysis and clinical management of pancreatic cancer cases.** (A) Mutational spectrum of 97 pancreatic cancer (PCA) cases grouped by pathogenic germline variant (PGV) status (left: PGV^−^, right: PGV^+^). PCA risk genes are shown in bold. Alterations marked as both ‘multihit’ and as PGV represent loss of heterozygosity (LOH). (B) Therapy recommendations supported by PGVs in the MASTER cohort. (C) Overview of cascade testing of relatives of individuals with PGVs. AD, autosomal dominant, AR, autosomal recessive; CNV, copy number variation; CPG, cancer predisposition gene; MMR, mismatch repair; MTB, molecular tumor board.

Germline analysis identified 33 PGVs across 14 CPGs, predominantly in 8 PCA risk genes [72.7%; *n* = 24/33 of PGVs: *BRCA1/2* (*n* = 5 each), *ATM* (*n* = 4), *PALB2* (*n* = 4), *MLH1* (*n* = 2), *MUTYH* (*n* = 2), *MSH2* (*n* = 1), *CDKN2A* (*n* = 1)]. Remaining PGVs occurred in six non-PCA risk genes [27.3%; *n* = 9/33: *BARD1* (*n* = 1), *CHEK2* (*n* = 2), *SDHB* (*n* = 1), *WT1* (*n* = 1, all autosomal dominant), *NBN* (*n* = 3), *BLM* (*n* = 1, both autosomal recessive)], with four patients harboring multiple autosomal dominant CPG variants (Figure 3A). There was no indication of mosaicism in analyzed genes in our cohort. However, copy number variation calling using panel data in the Dresden cohort was limited, and low-level mosaicism or copy number events below the detection threshold cannot be fully excluded.

Tumor sequencing data (available for 88/125 cases, 70.4%) revealed frequent and PCA characteristic somatic alterations in *KRAS* (75.0%, *n* = 66/88), *CDKN2A* (65.9%, *n* = 58/88), *TP53* (65.9%, *n* = 58/88), and *SMAD4* (60.2%, *n* = 53/88, Figure 3A). Technical challenges (e.g. poor DNA quality or low tumor cell content) limited data completeness in 16.8% of cases (*n* = 21/125). No somatic data were available in 13/125 cases (10.4%).

In the multidisciplinary molecular tumor board, 69.2% of PGV-positive patients (*n* = 9/13) received therapy recommendations, primarily PARP inhibitors (*BRCA1/2* alterations), immune checkpoint inhibitors, or CDK4/6 inhibitors (Figure 3B).^25^^,^^35^ Cascade testing of 34 relatives from 11 of 15 Dresden cohort patients with PGVs (73.3%) detected PGVs in 18 relatives (52.9%) from 7 different families, prompting tailored surveillance (Figure 3C).

### Broad panel sequencing of PCA patients from 11 different studies yields 6.7%-21.6% of patients with PGVs in autosomal dominant CPGs

**Figure 4:**
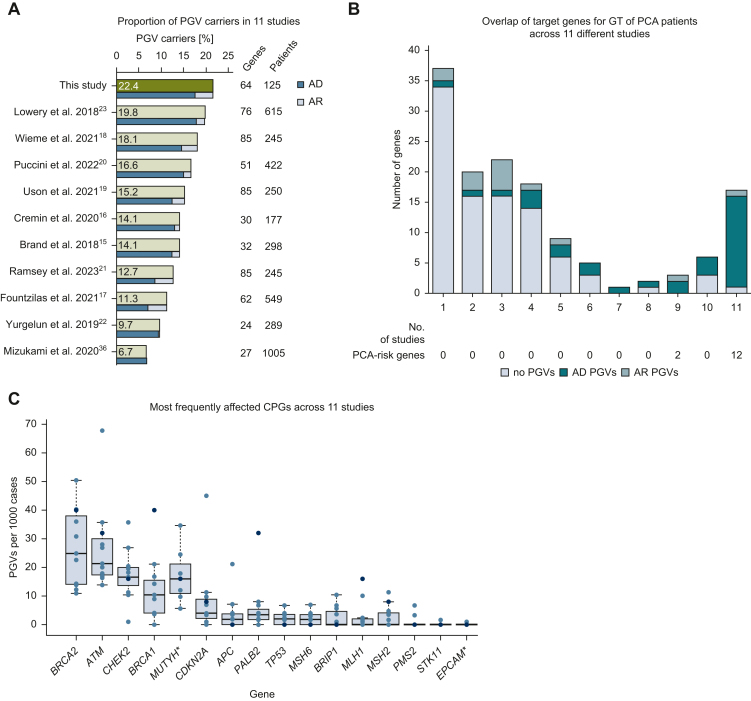
**Meta-analysis of pathogenic germline variants (PGVs) in pancreatic cancer across 11 studies.** (A) Percentage of pathogenic germline variant (PGV) carriers identified in different cohorts (green bars) in autosomal dominant (AD) cancer predisposition genes (CPGs) (blue bars) and autosomal recessive (AR) CPGs (Light blue bars). (B) Overlap of target genes across 11 studies with germline sequencing of pancreatic cancer (PCA) patients. Numbers of studies that nominated a specific gene and the PGV counts in AD and AR CPGs is depicted. (C) Most frequently affected CPGs across 11 studies, including PGV yields in 14 PCA risk genes (bold). Blue dots: this study. ∗*MUTYH* was analyzed in nine studies and heterozygous PGVs reported in seven studies. *EPCAM* was analyzed in nine studies. GT, genetic testing.

Analysis of 11 studies (including ours) with 125-1005 PCA patients (mean 387) assessed 140 CPGs (24-84 per study), revealing PGV yields of 6.7%-21.6% (mean 14.6%, median 14.1%, Figure 4A, Supplementary Table S3, available at https://doi.org/10.1016/j.esmogo.2025.100218).^23^^,^^36^ Among 18 CPGs analyzed in all studies (12 PCA risk genes), 80.3% of PGVs (*n* = 468/583) were detected in 11% of patients (Figure 4B). *BRCA2* (19.9%), *ATM* (16.6%), *CHEK2* (11.0%), and *BRCA1* (8.4%) were most frequent (Figure 4C). *MUTYH* and *EPCAM* were analyzed in 9/11 studies. *MUTYH* had high yields in two studies, though inconsistent reporting of monoallelic variants limited comparisons.^17^^,^^18^^,^^20^, ^21^, ^22^^,^^36^ Our cohort showed elevated *BRCA1*, *PALB2*, and *MLH1* PGV rates, potentially due to smaller size or enrichment for HBOC/LS families.

## Discussion

This study detected (likely) PGVs in 21.6% (17.6% autosomal dominant) of PCA patients, exceeding previously reported yields (5%-10%).^11^^,^^13^ This higher prevalence likely reflects cohort inclusion biases: Dresden cohort patients were primarily referred based on personal/familial cancer history (nearly half had a first-degree relative with PCA), while MASTER cohort patients were selected for advanced cancers diagnosed before age 51 years. Consistent with referral patterns, autosomal dominant CPG PGVs were significantly more frequent in the Dresden cohort (31.7%, *n* = 13/41) than in MASTER (10.7%, *n* = 9/84), independent of age. A meta-analysis of 11 studies (4264 PCA patients) revealed a median PGV yield of 14.1% across 140 CPGs.

Current guidelines (ASCO, ESMO, NCCN) show inconsistencies in testing criteria and gene panels. ESMO’s recommendation to test metastatic PCA patients for *BRCA1/2* variants risks missing other autosomal dominant CPG PGVs in cohorts enriched for FPC. Restricting testing to patients meeting FPC, LS, or HBOC criteria would have missed 23.8% of PGVs in our cohort. Among 21 autosomal dominant CPG PGVs detected in 64 patients, only 4 (19%) occurred in individuals meeting FPC criteria (*BRCA1*, *PALB2*, *ATM*, *CHEK2*).

Guideline discrepancies extend to gene selection, with variations in recommendations for *APC*, *CDK4*, *MUTYH*, and chronic pancreatitis genes.^10^^,^^11^^,^^13^^,^^34^ Limiting testing to 14 PCA risk genes would have missed 27%-28% of PGVs in this cohort. For example, *CHEK2*—a gene frequently altered in PCA (second to *BRCA2* and *ATM* in 4264 patients across 11 cohorts)—is excluded from many panels.^19^, ^20^, ^21^, ^22^, ^23^, ^24^, ^25^, ^26^, ^27^^,^^33^^,^^37^ Notably, among 29/64 patients (45.3%) not meeting FPC/LS/HBOC criteria, 4 (13.8%) harbored 6 autosomal dominant CPG PGVs (*BRCA2, BARD1, WT1, SDHB*, 2x *PALB2*), and 2 had *NBN* variants. While not part of international guideline recommendations, *BARD1* and *WT1* are recommended to be included for germline testing in the German S3-Guideline for exocrine PCA. The contribution of *SDHB* to PCA is unclear, but in our cohort, the PGV in *SDHB* occurred in a patient that also had a PGV in *PALB2*, which has a clear PCA association. However, *SDHB* is included in the ACMG recommendations for secondary findings and was thus included in the report.^38^ Strict adherence to guidelines would have denied four patients a hereditary cancer diagnosis and five patients the opportunity for targeted therapies (e.g. PARP inhibitors for *BRCA2/PALB2* alterations, immune checkpoint inhibitors for an microsatellite instability high case with *MSH2* PGV, CDK4/6 inhibitors for CDKN2A).^5^^,^^35^ The benefit of PARP inhibitors for non-*BRCA* DNA repair genes or autosomal recessive CPG carriers remains unclear.^5^^,^^6^

These findings underscore the underestimation of genetic risks in PCA and advocate universal large-panel germline testing, given the broad spectrum of implicated genes.^15^, ^16^, ^17^, ^18^, ^19^, ^20^, ^21^, ^22^, ^23^^,^^36^ Harmonizing guidelines to include all actionable CPGs—irrespective of established PCA associations—is critical. Where tumor-only sequencing is used, germline confirmation should be prioritized.^5^^,^^25^^,^^39^^,^^40^

Despite the considerable benefits of broad genetic testing, challenges remain in integrating this approach into routine clinical practice. Cascade testing in 73.3% of patients identified PGVs in 47.4% of relatives, aligning with reports from centers using universal testing.^37^^,^^41^ Barriers include advanced disease, older age, and lack of social support.^37^ NCCN emphasizes rapid germline testing due to PCA’s poor prognosis, yet 16 families were excluded when index patients died before testing.^10^ Among these, six families had PGVs (*BRCA2*, *BRCA1*, *CDKN2A, CHEK2, NBN*). In one case, a *CDKN2A* PGV was identified in a relative only after a melanoma diagnosis (and the subsequent diagnosis of FAMMM), highlighting missed opportunities when providers deny testing due to restrictive criteria. Another important factor is the cost-effectiveness of universal germline testing. A 2020 economic evaluation comparing population-based *BRCA1/2* testing with clinical criteria-based testing for HBOC demonstrated that population-based testing is cost-saving in high-income countries (e.g. UK, USA) and cost-effective in upper-middle-income countries (e.g. China, Brazil).^42^ However, it was not found to be cost-effective in low-middle-income countries such as India unless the cost of testing is substantially reduced.^42^ These findings highlight the sensitivity of cost-effectiveness outcomes to the price of genetic testing and underscore the complexity involved in generalizing such conclusions across different settings. Importantly, these results indicate that universal germline testing in PCA patients may be economically justifiable and feasible, supporting its potential value also from a health economics standpoint.

While the presence of PGVs in PCA patients can inform clinical decisions, PGV interpretation remains complex, particularly for variants in understudied genes. At least 30% of families, especially in the Dresden cohort, have relatives eligible for PCA screening, necessitating multidisciplinary collaboration among geneticists, pathologists, and oncologists.

Our findings suggest that PGVs are more prevalent in PCA than historically recognized. Genetic testing should follow ASCO/NCCN guidelines and expand to all PCA patients, regardless of age or family history. Standardized, comprehensive panels are essential to optimize diagnosis, therapy, and cascade testing. Integrating genetic data into precision oncology is not merely advantageous but imperative for improving outcomes. A study showed significant survival benefits for patients with PGVs in *CDKN2A* undergoing surveillance for pancreatic cancer compared with those not in surveillance.^43^ Surveillance led to earlier detection, increased resectability, and a 5-year survival rate of 32.4% compared with 4.3% for nonsurveillance patients, supporting the notion that early detection strategies—including germline testing—can be life-saving when paired with high-quality, guideline-based care in certified centers to ensure low complication rates and the best possible results for all patients.^43^

## Conclusions

PGVs are under-recognized in PCA. Harmonized guidelines should mandate comprehensive gene panels (beyond *BRCA1/2*) for all patients, irrespective of age/family history, to optimize diagnosis, therapy, and cascade testing. Multidisciplinary collaboration is essential to address clinical complexities and improve outcomes.

## Declaration of generative AI and AI-assisted technologies in the writing process

During the preparation of this work the authors used Perplexity AI in order to refine phrasing and grammar. After using this tool, the authors reviewed and edited the content as needed and take full responsibility for the content of the publication.

## Disclosure

Consultancies: **AS**: Aignostics, Amgen, Astra Zeneca, Bayer, BMS, Eli Lilly, Illumina, Incyte, Janssen, Jazz Pharmaceuticals, Merck, MSD, Novartis, Pfizer, Roche, Servier, Sanofi, Takeda, and Thermo Fisher. **TH**: Servier and Jazz Pharmaceuticals. **SF**: Illumina. Honoraria: **TH**: Astellas Pharma GmbH. Grants or other funding: **AS**: Bayer, BMS, MSD, Chugai, and Incyte. **TH**: Roche.

All remaining authors have declared no conflicts of interest.

## Supplememtary data

Supplememtary table
